# White-crested elaenias (*Elaenia albiceps chilensis*) breeding across Patagonia exhibit similar spatial and temporal movement patterns throughout the year

**DOI:** 10.1371/journal.pone.0299954

**Published:** 2024-04-18

**Authors:** Rocío Fernanda Jara, Jaime Enrique Jiménez, Ricardo Rozzi

**Affiliations:** 1 Department of Biological Sciences, University of North Texas, Denton, TX, United States of America; 2 Sub-Antarctic Biocultural Conservation Program, University of North Texas, Denton, TX, United States of America; 3 Cape Horn International Center (CHIC), Puerto Williams, Magallanes, Chile; 4 Advanced Environmental Research Institute, University of North Texas, Denton, TX, United States of America; 5 Universidad de Magallanes, Puerto Williams, Chile; 6 Department of Philosophy and Religion, University of North Texas, Denton, TX, United States of America; National Cheng Kung University, TAIWAN

## Abstract

For migratory birds, events happening during any period of their annual cycle can have strong carry-over effects on the subsequent periods. The strength of carry-over effects between non-breeding and breeding grounds can be shaped by the degree of migratory connectivity: whether or not individuals that breed together also migrate and/or spend the non-breeding season together. We assessed the annual cycle of the White-crested Elaenia (*Elaenia albiceps chilensis*), the longest-distance migrant flycatcher within South America, which breeds in Patagonia and spends the non-breeding season as far north as Amazonia. Using light-level geolocators, we tracked the annual movements of elaenias breeding on southern Patagonia and compared it with movements of elaenias breeding in northern Patagonia (1,365 km north) using Movebank Repository data. We found that elaenias breeding in southern Patagonia successively used two separate non-breeding regions while in their Brazilian non-breeding grounds, as already found for elaenias breeding in the northern Patagonia site. Elaenias breeding in both northern and southern Patagonia also showed high spread in their non-breeding grounds, high non-breeding overlap among individuals from both breeding sites, and similar migration phenology, all of which suggests weak migratory connectivity for this species. Elucidating the annual cycle of this species, with particular emphasis on females and juveniles, still requires further research across a wide expanse of South America. This information will be critical to understanding and possibly predicting this species’ response to climate change and rapid land-use changes.

## Introduction

The annual cycle of birds that migrate between temperate and tropical regions have been studied almost exclusively in the northern hemisphere (e.g., [1]). Filling this gap is imperative given the marked contrast between the northern and southern hemispheres in attributes such as the ratios of tropical and temperate forests and the shapes of the land masses [2,3]. Unlike the extensive temperate and boreal forests in the northern hemisphere, in South America, tropical forests are extensive but temperate forests are limited to a narrow band in southern Chile and western Argentina. Therefore, South America presents the inverse situation to North America with respect to the extent of habitat and geographic area during the breeding and non-breeding seasons of birds. This offers a unique opportunity to investigate the connectivity between non-breeding (tropical region) and breeding (temperate region) regions in a still understudied geographical region.

Migratory connectivity exists as a continuum from strong to weak [4], and is influenced by both a spatial and temporal component [5]. During the stationary periods (i.e., breeding and non-breeding), migratory connectivity is mostly influenced by the spatial distribution of individuals [5]. Strong spatial connectivity occurs when most individuals from a breeding population or site spend the non-breeding season together in the same region [4,6]. For example, Golden-winged Warblers (*Vermivora cyanoptera*) breeding in the Great Lakes region spend the non-breeding season in Central America, whereas Golden-winged Warblers breeding in the Appalachian Mountains spend the non-breeding season in a separate region in northern South America [7]. In contrast, weak spatial connectivity occurs when there is no spatial segregation among individuals and, for example, individuals from different breeding populations co-occur in the same non-breeding sites [8]. For example, Purple Martins (*Progne subis subis*) breeding in far distant and different habitats such as British Columbia, Minnesota and Texas, co-occur in northern Brazil near the Amazon river during their non-breeding season [9]. During the migratory periods of the annual cycle migratory connectivity is also influenced by the temporal arrangement of individuals according to their breeding origin [6]. Temporal connectivity is strong when there is asynchrony in migration between individuals from different breeding origin. That is, individuals from the same breeding origin depart from/arrive to the breeding or non-breeding regions around the same time, without temporally overlapping with individuals from different breeding origins. Under this scenario, even if a stopover site or non-breeding region is used by individuals from multiple breeding origins, they use the site at different times. Conversely, temporal connectivity is weak when there is no temporal segregation of individuals depending on their breeding origin (i.e., there is temporal overlap among individuals from different breeding origins at a given stopover or non-breeding site) [5].

For migratory birds, there is a link between the non-breeding and breeding regions, such that events happening on the non-breeding region can have strong carry-over effects on their subsequent breeding success [10–14]. If the connectivity is strong, carry-over effects can impact the population dynamics of a species, with the strength of carry-over effects being shaped by the degree of migratory connectivity [4,15]. For example, female American Redstarts (*Setophaga ruticilla*) that spend their non-breeding season in high-quality areas produce more chicks and their offspring fledge earlier than females spending their non-breeding season in low-quality areas [16]. This can have consequences on productivity and population dynamics for this species [16]. Thus, under a strong connectivity scenario, local habitat disturbance on the non-breeding grounds could have strong consequences on population recruitment in a specific breeding area [4]. In contrast, if individuals of a breeding population use separate non-breeding sites, the effect of local habitat disturbance in one of their non-breeding sites could have a weaker effect on their population dynamics [8].

In this study we assessed White-crested Elaenia’s (*Elaenia albiceps chilensis*; hereafter ‘elaenia’) movements during the annual cycle. This small (approximately 16-g) flycatcher is one of the longest-distance migrant birds within South America [17,18]. It migrates yearly between its non-breeding grounds in northeastern and central South America and its breeding grounds in the temperate forests of southern Chile and Argentina [18,19]. Elaenias breed from Copiapó, Chile (29°S) [20] to the southernmost forests of the world within the Cape Horn Biosphere Reserve (56°S) [21,22].

We built on previous research that studied the annual cycle of elaenias breeding at a site in northern Patagonia [19]. We collected similar data at a site in southern Patagonia and combined data from the two sites to evaluate the annual cycle of elaenias breeding across Patagonia. First, we evaluated whether elaenias breeding at the southern Patagonian site utilize specific regions sequentially throughout the non-breeding season, as was found for eleanias breeding in northern Patagonia [19]. Second, we assessed the level of migratory connectivity of elaenias breeding across Patagonia, looking at the spatial and temporal arrangement of individuals during the non-breeding season. Spatially, we predicted that elaenias breeding in southern Patagonia would exploit a vast area within the non-breeding region, as was found—although not quantitatively measured—for elaenias breeding in northern Patagonia [19]. Temporally, we predicted that the phenology of migration should be delayed at the southern relative to the northern site, as has been found in other long-distance migrants and attributed to the delayed availability of resources on the breeding grounds at higher latitudes [23–25].

## Materials and methods

### Study sites

**Fig 1:**
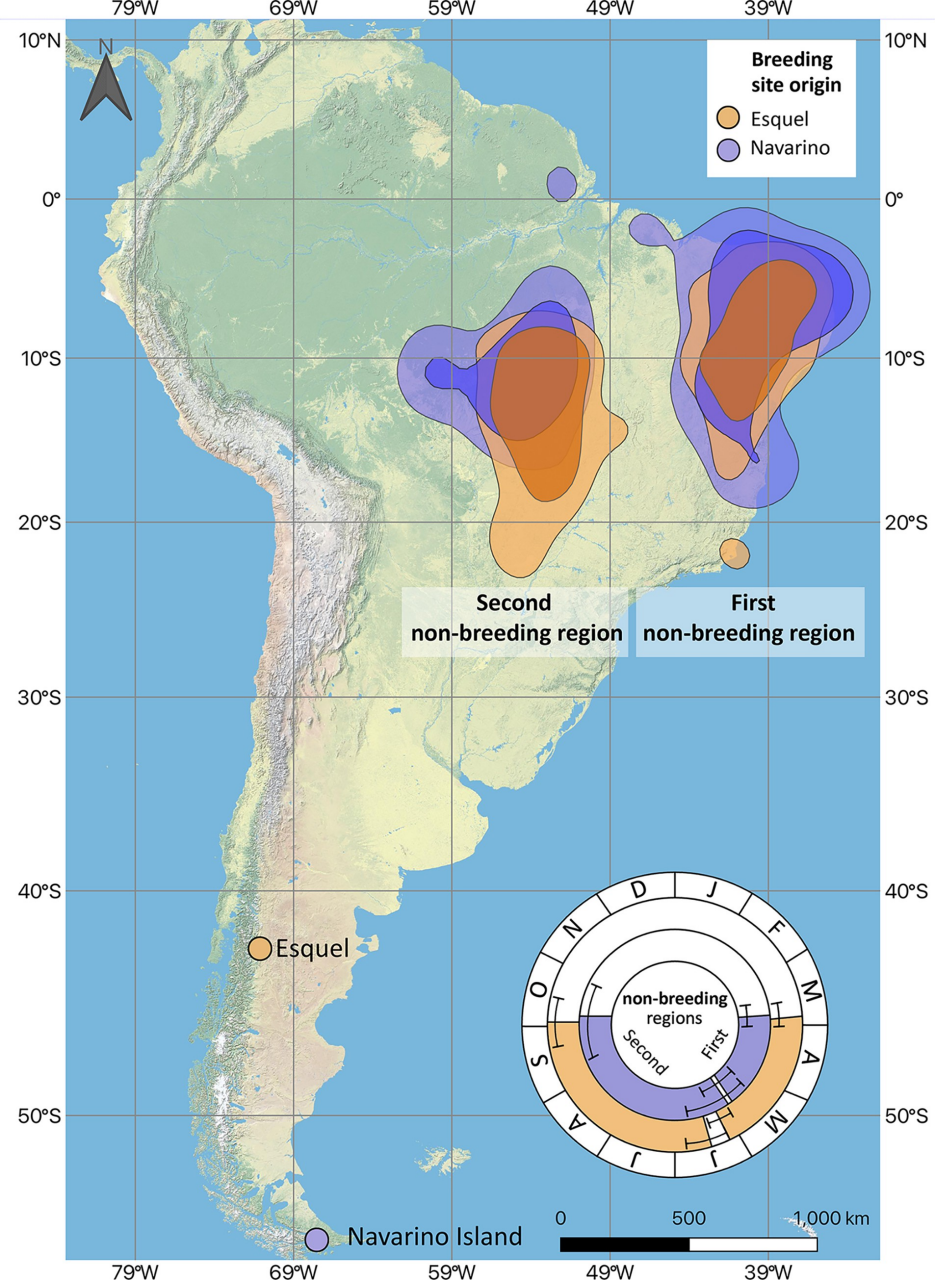
Non-breeding regions for *Elaenia albiceps chilensis* breeding in Esquel and Navarino Island. The polygons depict the 50% (darker shade) and 75% (lighter shade) kernel density utilization distribution of all the points during the non-breeding periods across individuals. Data of Esquel breeding site obtained from Movebank Data Repository [28]. The wheel inset represents temporal utilization of each non-breeding region by breeding site origin. The limits of the colored area represent the average date and the bars extending from it their 95% Confidence Interval. Figure design and assembly by Mauricio Alvarez. Base map and data from OpenStreetMap and OpenStreetMap Foundation.

We worked at Navarino Island, which is embedded in the world’s southernmost forests (Fig 1), where since 2000, the southernmost breeding population of elaenias have been monitored at the Omora Ethnobotanical Park on Navarino Island (54°S) within the Cape Horn Biosphere Reserve, Chile [26]. We concentrated our efforts in the more accessible forests of Omora Ethnobotanical Park (54°56′ S, 67°39′ W) and immediately surrounding areas. The forests here are dominated by two deciduous species (*Nothofagus antarctica* and *N*. *pumilio*) and one evergreen species (*N*. *betuloides*). The understory is composed of low trees (*Drimys winteri*) and shrubs (e.g., *Berberis buxifolia*, *B*. *ilicifolia*, *Ribes magellanicum*) as well as *Embothrium coccineum* [27]. We also built on previous research for which we accessed data publicly archived in the Movebank Data Repository [28], collected at a second site located 1,365 km to the north of Navarino Island, in the temperate forests of northwestern Patagonia at the "Cañadón Florido" cattle ranch (42°55’S, 71°21’W), near Esquel, Province of Chubut, Argentina (hereafter “Esquel”) [19]. The forests here are dominated by *Maytenus boaria*, *Schinus patagonicus* and *Nothofagus antarctica* [19,29].

### Bird capture and geolocator deployment

On Navarino Island, during two breeding seasons (2014–15 and 2015–16), we captured individuals with mist nets of mesh size 30 mm and 12 m long and the help of a decoy and broadcasting of conspecific calls and songs. Once captured, we determined the individual’s age, weight, sex, and breeding status based on size of cloacal protuberance or brood patch development. We banded each captured bird with a Chilean Agriculture and Livestock Service (SAG, by its acronym in Spanish) aluminum band. We also added three plastic color leg bands to facilitate future identification of individuals carrying a geolocator. This way, during subsequent breeding seasons, we only attempted to recapture individuals known to have a geolocator. We deployed 117 light-level geolocators (Intigeo P55B1-7, Migrate Techonology, Ltd, Coton, Cambridge, UK) on breeding/post breeding adults. The geolocators were attached using a leg-loop harness [30]. Teflon ribbon or elastic nylon cord (Bead‘N Stretch brand, 0.7 mm diameter), depending on availability, was used to make the harness. One of these geolocators recorded light data for two full annual cycles, but we only analyzed the data from the first annual cycle, to maintain consistency among all samples. Similarly, on Esquel, 45 adult elaenias were fitted with the same model of geolocators that we used at Navarino Island (see [19] for specific details concerning field methods). Geolocators deployed at both sites measured light levels every minute and recorded maximum light level every 5 min.

Of the 117 individuals fitted with geolocators from Navarino Island, 57.3% (*n* = 67) were males and 31.6% (*n* = 37) were females. We were unable to determine the sex of the remaining 11.1% individuals (*n* = 13). After quick processing (max. 15 min), we released the individuals in the same capture location and monitored each bird for 1–3 minutes after release. None of the geolocators interfered with the bird’s ability to fly. The average weight of the geolocators was 0.6 g, (including the harness), representing 3.76% ± 0.44 (mean ± 1SD, *n* = 10) of the bird’s body weight.

During the subsequent breeding season (the season after the non-breeding period), we visually searched for color-banded individuals in the areas where we captured and marked birds. Once re-sighted, we recaptured and recovered geolocators following the same capture protocol described above. None of the recaptured individuals had signs of abrasions or lesions associated with the geolocator. Although we recaptured all the individuals fitted with a geolocator that we detected at the study site, these individuals were considerably less responsive to the decoy and playback than non-banded birds, which required additional efforts to recapture them.

Protocols to handle our study animals and attach geolocators using a leg-loop harness were approved by SAG (permit numbers 1058/2014, 300/2015 and 5158/2016) and no further action was required by the AICUC committee at the University of North Texas. All methods were performed in accordance with the guidelines to the use of wild birds in research [31].

### Recovered geolocators on Navarino Island breeding site

At Navarino Island, we recovered a total 12 of the 177 geolocators deployed, six from each of the two breeding seasons (10.5% and 10.0% recovery rate for the first and second season, respectively). We recovered eleven geolocators from males (*n* = 67, 16% recovery rate) and one from a female (*n* = 37, 3% recovery rate), which resulted in higher recovery rates for males ($X^{2}$ = 4.393, df = 1, p-value = 0.036). All 12 geolocators were recovered <1 km from the location where we deployed them, further supporting previous studies suggesting that adult elaenias have high inter-annual site fidelity [32]. We recaptured all birds that were visually identified as fitted with a geolocator. All retrieved geolocators were not recording data upon arriving to Navarino Island, providing incomplete light intensity data for the full annual cycle. Thus, we shipped them to Migrate Technology to download each tag’s data. Furthermore, two of the geolocators recorded light data that were strongly affected by shading, preventing the use of curve-fitting analysis methods. Therefore, we did not include those two geolocators in our analysis, and we only analyzed data recorded from the remaining ten recovered geolocators. We complemented our data with five geolocators: three recovered from a previous pilot study and two recovered as part of long-term migration studies at Omora Park. Details on the numbers of geolocators deployed and recovered per breeding season are provided in S1 Table. About 50% of the geolocators (n = 8) stopped recording data when the birds were moving from the first to the second wintering region (See S2 Table). Of the remaining seven geolocators, four recorded data during spring migration, with only two of them recorded data for the full annual cycle (See S2 Table and S1 Fig). Overall, we present data from 14 adult males and one adult female for Navarino Island.

We combined raw geolocator data from geolocators deployed and recovered on Navarino Island (n = 15), Chile, with the published data of geolocators deployed and recovered in Esquel, Argentina [28]. In Esquel, Bravo *et al*. [19] recovered 15 (14 males and one female) of 45 geolocators deployed at their breeding site. Most of the publicly available data archived in Movebank include light records of individuals from their breeding season through the initiation of their spring migration (n = 12), but only seven tags recorded light intensity for the complete annual cycle [28]. See details in Bravo et al. ([19]; S1 Table). Overall, we analyzed combined data for 30 recovered geolocators (from both Navarino Island and Esquel) using the methods described below.

### Data analysis

For all data processing and location estimation, we used custom open-source tools for geolocator-analysis in R (R version 3.5.2; [33]). We annotated twilight events using the “preprocessLight” function in TwGeos package with a threshold light level value of 1. We removed aberrant twilight events manually with the “preprocessLight” function. On average, we removed 5.09 ± 3.01% (*n* = 15) of the twilight events for Navarino, and 2.18 ± 1.97% (*n* = 15) for Esquel. We calibrated the geolocators on the birds while on the breeding grounds (i.e., this was the known location) using the “make.calibration” function in FLightR package [34,35]. To derive the period that the birds stayed on the breeding grounds, we used the “plot_slopes_by_location” function in the FLightR package.

To estimate locations based on light intensity data, we used a particle filter in FLightR package. FLightR uses a hidden Markov chain model to estimate a probability distribution of the bird’s location, which produces the most likely migration route based on light-level data. Because elaenias are known to inhabit islands (e.g., Navarino Island), in our model we allowed birds flying over oceanic water (up to 1000 km from the coast) but limited birds’ stationary periods to land only. We used the “stationary.migration.summary” function in FLightR (with a 0.3 probability of movement) to estimate stationary periods (stopover, non-breeding and breeding sites) and to estimate when birds arrived and departed from those sites. We defined a given non-breeding site as a stationary period that lasted at least 20 days. We chose that cut-off value as there was a natural break in the duration of stationary period data (i.e., there was a bimodal distribution of stationary period length, where most periods lasted either well under or over 20 days). Results were similar when we used a cut-off value of 30 days, which was the value used in another study investigating migration patterns with another migrant flycatcher in South America (*Tyrannus savana*) [23]. We represent non-breeding sites as the median longitude and latitude for the given stationary period, and error is given by their first and third quartiles (see S1 Fig, the polygons are formed by the connection of first and third quartiles for median longitude and latitude of each non-breeding site). We defined fall migration as the period between the date when a bird left the breeding site and the date of the first stationary period that lasted at least 20 days. Similarly, we defined initiation of spring migration as the first day of movement after a stationary period of at least 20 days that was not followed by another stationary period of at least 20 days. Termination of spring migration occurred on the day the birds arrived in the area where they were captured the previous year during their breeding period.

Migratory connectivity patterns include two key components: population spread and inter-population mixing [8]. Population spread describes how much a single population spreads out across the non-breeding grounds [8]. Inter-population mixing can be defined as the degree of co-occurrence of individuals from different populations on the non-breeding grounds [8]. In this study we estimated migratory connectivity for a single population, using individuals sampled at different locations within the breeding range. Therefore, the spread and intermixing we report here are site-based, rather than population-based, because we do not have evidence that the sites represent separate populations.

To describe spread on the non-breeding grounds, we estimated the pairwise distance among individuals. We calculated distances based on the median longitude and latitude of each individual’s non-breeding sites using the Alteryx software version 2020.2.3.27789 (Irvine, CA, USA). To visualize the spread and quantify their overlap during their non-breeding period, first we estimated the 50% and 75% kernel density utilization distribution (kernel UD) of all the points during the non-breeding period of each individual, using the “adehabitatHR” package [36–38].

Our finding of two large non-breeding regions used by elaenias from both sites (see Results) demanded additional analyses to explore the spread for each region. For the first non-breeding region we obtained data from 15 individuals from each breeding site (Navarino and Esquel). However, because the battery of some geolocators ran out, for the second non-breeding region we were able to record data only for seven individuals of Navarino and 12 of Esquel. Furthermore, one of the seven geolocators from Navarino stopped recording data before the individual departed from the second non-breeding region. The amount of overlap of non-breeding regions between elaenias from the two breeding sites was estimated using the “kerneloverlaphr” of the “adehabitatHR” package with the “HR” method.

We quantified the strength of migratory connectivity (proximity of elaenias to each other on the non-breeding grounds according to their breeding origin) with the Mantel correlation coefficient (*r*_M_) [39]. This coefficient is a measure of the correlation between two matrices representing the geographic distances among individuals in both the breeding and non-breeding grounds. A strong positive correlation between the two matrices results when individuals breed and also spend the non-breeding season in close proximity to each other (strong migratory connectivity). Conversely, a weak or negative correlation results when individuals that breed close together spend the non-breeding season in different locations (weak connectivity; [39]). Because elaenias from both breeding sites used two non-breeding regions sequentially (see Results), we tested the strength of migratory connectivity, considering both non-breeding regions separately. To estimate the distance matrices used for the Mantel test, for each non-breeding region we combined data of individuals from both breeding sites (i.e., *n* = 30 for the first non-breeding region and *n* = 19 for the second one). For elaenias that had more than one stationary period within a non-breeding region (S2 Table), we used the first period only.

We also assessed temporal migratory connectivity between individuals from both breeding sites by testing for differences in arrival and departure dates for both non-breeding regions separately using a Wilcoxon Rank Sum test.

## Results

### General migration patterns

**Fig 2:**
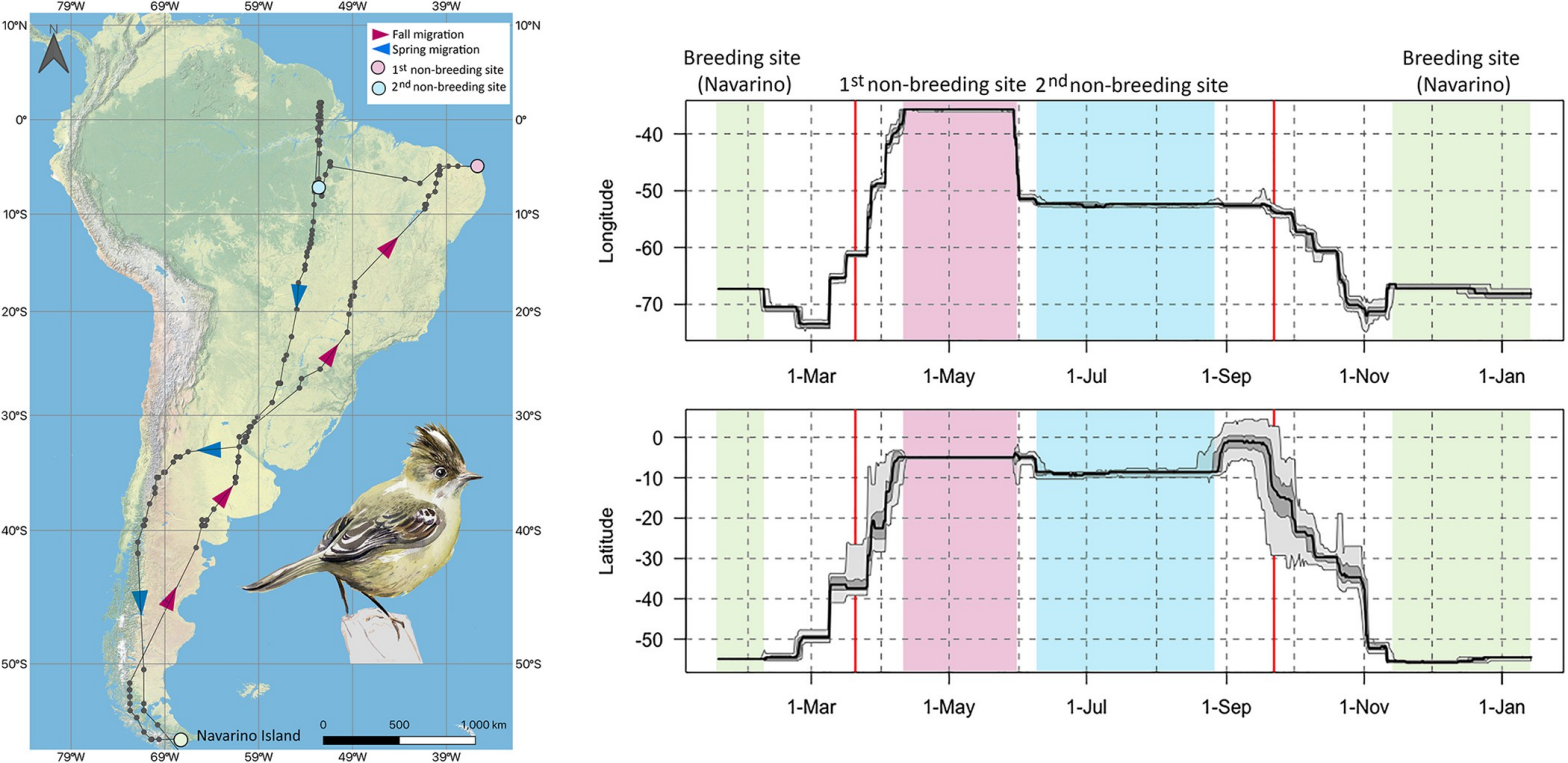
Annual movements (an example of an individual) of *Elaenia albiceps chilensis* breeding on Navarino Island. Dark dots connected by lines in the figure on the left represent the median of estimated locations for each twilight event. Triangles placed over the line indicate the direction of movement during Fall and Spring migration. Figures on the right column show changes in estimated longitude (top panels) and latitude (bottom panels). The black lines represent the median of the estimated location; dark shading represents the interquartile range and light gray shading the 95% credible interval. Red vertical lines represent equinoxes [35]. Figure design and assembly by Mauricio Alvarez. Bird illustration by Claudia Blin; used with permission by the artist. Base map and data from OpenStreetMap and OpenStreetMap Foundation.

During their fall migration, 87% (13 of 15) of the tracked elaenias from Navarino Island migrated northwards along the Andes Mountain range, through temperate forests habitats (Fig 2 and S1 Fig), moving through the breeding range for this subspecies [20]. The other two elaenias took a different route: one flew north along the Atlantic coast, and the other through the Patagonian steppe (S1 Fig). Between 45 and 37°S, the 13 elaenias that migrated along the Andes flew east crossing the Arid Steppe, and then the Humid Pampas, Espinal, and Low Monte ecoregions [40] in Argentina, reaching the Bahia Interior and Coastal Forests along the Atlantic at approximately 10°S in Brazil (Fig 2 and S1 Fig). The tracked elaenias then migrated north until reaching the Caatinga and Bahia Coastal Forest ecoregions in Brazil, where all of them spent the first part of the non-breeding season (first non-breeding region) before migrating westward to central Brazil (second non-breeding region).

For the four individuals for which we have partial (H795 and BC037) and full (H796 and BJ354) spring migration data, most of the tracked elaenias migrated south through the Dry and Humid Chaco in Paraguay and Argentina, and then southwest, crossing the Humid Pampas, Espinal, and Low Monte ecoregions in Argentina (S1 Fig). From there, the tracked elaenias followed a similar route as during fall migration, flying southwards along the Andes until arriving at Navarino Island (S1 Fig). Error estimates of the locations are shown in detail for each individual in S1 Fig.

### Migratory connectivity

Elaenias spent ~6.4 months in Brazil during their non-breeding season. During that period, elaenias from both sites sequentially occupied two distinct non-breeding regions (Fig 1). In each of these non-breeding regions they had 1–3 stationary periods in slightly different locations (i.e., non-breeding sites) but within the same general non-breeding regions in northeastern and central Brazil. The first non-breeding region was in northeastern Brazil, where most individuals stayed in the Caatinga dry forest ecoregion (Fig 1). The Bahia Coastal forests were used to a lesser degree (S1 Fig). The second non-breeding region was in central Brazil, were most elaenias (77%) stayed in the Mato Grosso seasonal forest and the tropical savanna of the Cerrado ecoregion (Fig 1).

Mantel tests indicated weak spatial migratory connectivity for both the first (*r*_M_ = -0.017, *P* = 0.621, *n* = 30) and second (*r*_M_ = 0.096, *P* = 0.280, *n* = 19) non-breeding regions; individuals that bred together did not spend the non-breeding season together. The spread in the two breeding sites was very small, less than 1.5 km in each site. This small distancing among individuals captured at the two breeding sites contrasts sharply with the distance held among the same individuals during their stay in the non-breeding sites. While almost all individuals from both breeding sites generally used the same first and second non-breeding regions, spread in the non-breeding grounds encompassed up to thousands of kilometers. For the first non-breeding region, individuals from Navarino used sites that were on average 740 km apart (95% CI = 660–819, maximum distance = 1606 km, *n* = 15), and those from Esquel 633 km apart (CI = 551–715, max = 2102, *n* = 15) (Fig 1). For the second non-breeding region, individuals from Navarino used sites that were 466 km apart (CI = 360–572, max = 936, *n* = 7), and those from Esquel 483 km apart (CI = 416–550 km, max = 1228 km, *n* = 12) (Fig 1). Moreover, there was considerable overlap between the non-breeding regions used by the two groups, with 47% overlap in the non-breeding region use by elaenias from Navarino Island with those from Esquel (or 40% overlap by elaenias from Esquel with those from Navarino Island) (Fig 1). The observed overlap is larger when considering only the first non-breeding region in northeastern Brazil, with 85% overlap by elaenias from Esquel with those from Navarino Island (Fig 1).

We found mostly weak temporal migratory connectivity, with only one difference detected in departure/arrival patterns from/to both non-breeding regions. Specifically, there was no difference in arrival date at the first non-breeding region between elaenias originating from both sites (March 25^th^ for Navarino [*n* = 15, 95% CI = March 17^th^–April 2^nd^] and March 24^th^ for Esquel sites [*n* = 15, CI = March 17^th^–April 1^st^]; *W* = 108.5, *P* = 0.884; Fig 1). However, individuals from Navarino departed on average from the first non-breeding region at an earlier date than elaenias from Esquel (May 26^th^ for Navarino [*n* = 14, CI = May 18^th^–June 4^th^] and June 6^th^ for Esquel [*n* = 15, CI = May 30^th^–June 13^th^] respectively; *W* = 225, *P* < 0.001; Fig 1). For the second non-breeding region, there were no differences in arrival and departure date between Navarino Island and Esquel elaenias (Arrival *W* = 65.5, *P* = 0.051; departure *W* = 37.5, *P* = 0.925). However, sample size was limited because a high proportion of the geolocators of birds tagged on Navarino Island (*n* = 8; 53%) were no longer recording data before they arrived at their second non-breeding region (S2 Table).

## Discussion

Every year, dozens of species migrate within South America as they track seasonally available resources and avoid harsh winters [41,42]. Among these, the White-crested Elaenia spends the non-breeding season in tropical forests and breeds in the temperate forests of southern South America [18,19,43].

Overall, we found that elaenias from both sites exhibited a similar pattern of sequentially using different non-breeding regions as found by Bravo *et al*. [19]. While in Brazil, they used non-breeding sites that were up to 2102 km apart (i.e., high spread), and the amount of overlap between individuals from the two sampled breeding sites was as high as 85% in the first non-breeding region (i.e., high inter-site mixing) (Fig 1), with no spatial correlation between the two groups, which indicates weak spatial migratory connectivity. Our findings contrast with a previous study on five Neotropical-Nearctic warblers that showed strong migratory connectivity in populations with high non-breeding spread [44]. It is unclear whether these contrasting findings represent a difference between the Neotropical-Nearctic and Neotropical austral migration systems in general, or merely variation among species in this regard.

We also found that elaenias breeding further south had a similar movement phenology to those breeding further north (i.e., weak temporal migratory connectivity), which did not support our prediction that elaenias breeding further south should exhibit a delayed schedule. We made this prediction based on the hypothesis that spring migration is delayed for higher-latitude breeders due to delayed resource availability on their breeding grounds, which in turn delays the entire annual cycle [25]. Spring arrival date of White-crested Elaenias breeding in central Chile [45] is indeed about a month earlier than those breeding on Navarino Island [21]. However, the start and peak of breeding activity occur on the same date in both sites [21]. The lack of delayed breeding timing on Navarino Island could explain why these individuals did not delay the start of their fall migration in contrast to other high-latitude breeders [24,25]. Although our sample sizes were limited, particularly for spring migration, which limits further exploration of the timing of migration, both temporal and spatial migratory connectivity appear to be weak: elaenias breeding together do not spend the non-breeding season together.

Our finding that elaenias from across Patagonia overlap extensively during the non-breeding season corresponds with those for long-distance migrant landbirds in general. Whinchats (*Saxicola rubetra*) from different parts of the breeding range overlapped across a wide non-breeding area in western Africa [46]. Moreover, the large non-breeding area used by elaenias may allow for increased migration adaptation potential. Even though elaenias spend the non-breeding season in the same ecoregions, they used different non-breeding sites within it (up to thousands of kilometers apart). Thus, elaenias may be under different selection pressures during the non-breeding season if they are exposed to different environmental conditions. If so, gene flow during the breeding season between individuals that spend the non-breeding season in different locations can allow for substantial genetic variability within each breeding site [47]. This is of crucial importance when considering the potential effect of non-breeding habitat loss in Brazil, because more genetic variability within each breeding site may allow for increased adaptation potential associated with migration, as a product of individuals migrating to different non-breeding sites.

The three ecoregions that the tracked elaenias occupied most frequently during the non-breeding season—Caatinga, Mato Grosso, and Cerrado—are currently under increasing pressure due to anthropogenic activities, and extensive areas of native vegetation in all three ecoregions have been cleared to allow for increased agriculture and cattle ranching [48,49]. The quality of non-breeding habitat ca have direct consequences on individual fitness via carry-over effects. For example, low quality non-breeding habitat may result in individuals having lower body condition at the end of the non-breeding season, resulting in a delayed spring migration and late arrival on the breeding grounds (e.g. [16,50,51]). Late arrival can result in a delay in the initiation of breeding (e.g. [52,53]), which can translate to lower reproductive success [16,54–56]. Due to the large non-breeding area used by the studied elaenias, habitat disturbances on their non-breeding grounds may have consequences that are widespread among breeding populations (see also [8]), but the severity of those consequences may differ among different breeding sites.

On Navarino Island we found significantly lower recapture rate for females fitted with geolocators than males (3% vs 16%, respectively). This low recapture rate for females was also lower than recapture rates reported by the Long-Term Ornithological Research Program at the Omora Ethnobotanical Park [32], which had a 17% recapture rate for adult elaenias of both sexes between 2005 and 2014 (not fitted with geolocators; unpublished data). Bravo *et al*. [19] also reported lower return rates (‘return rate’ = recapture plus re-sighting of color banded individuals) on Esquel female elaenias fitted with geolocators (25%) than fitted males (63%); however, the return rate of fitted females was higher than those without them. Fitted male return rate was the same as those without geolocators. (As on Navarino Island we recaptured all re-sighted color-banded individuals, our ‘recovery rates’ are equal to ‘return rates’). The lower recapture rate of fitted females we had on Navarino could be due to geolocators negatively affecting apparent survival of female elaenias, although it is not clear why this only affected females, nor why the pattern appears different from elaenias breeding at Esquel. The use of geolocators has been shown to have a small but significant effect on apparent survival of tracked birds [1,57]. Meta-analyses suggest that the negative effect on survival could be higher in smaller species, birds fitted with harnesses made of elastic material (instead of non-elastic material), and with higher geolocator load, although there is no consensus on geolocator load guidelines (our geolocator load, including the harness, was 3.76% of the body weight) [1,58,59]. Although we did not directly assess the potential effect of geolocators on elaenias’ apparent survival and other traits (e.g., body mass, reproduction, parental care, etc.), we cannot rule this out. While we consider that tracking elaenias (a species without conservation concern) with geolocators was ethical and provided valuable information on this species’ movement, future studies on this species should aim at using tracking devices that are as small as possible and fit them with non-elastic material [1].

Even though this study provides a comprehensive assessment of the annual cycle of *E*. *albiceps chilensis*, the longest-distance migrant flycatcher in South America, the results should be taken with caution as they mostly represent adult males and because the overall return rate for this species was low. Elaenias breeding in Patagonia had extensive spatial and temporal overlap during the non-breeding season, which included two separate non-breeding regions used in succession. This study raises new questions, such as why elaenias move to a second non-breeding region. We hypothesize they may be tracking resource availability (e.g., driven by patterns of temperature and precipitation), which would drive them from an area of declining resource availability to an area of greater available resources [60–62]. Alternatively, they could be tracking their local climate niche, like Yellow Warblers (*Setophaga petechia*) do across their annual cycle [63]. Further research is needed to understand the driving forces behind the movements of elaenias throughout their annual cycle. For example, evidence from congeners suggests that intra-specific genetic diversity is related to migration [64]. Furthermore, it is not known whether the migratory movements of these two breeding populations represent the movements of the whole subspecies, or whether elaenias breeding in other areas have different patterns. Elucidating the annual cycle of this species, with particular emphasis on females and juveniles, will require research across a wide expanse of South America, which will be critical to understand and possibly predict this species’ response to global change, particularly climate change [65] and rapid land-use changes that are taking place in South America at both temperate and tropical latitudes [66–69].

## Supporting information

S1 Fig Individual movement maps of elaenias breeding on Navarino Island (‘N’ in the ID) and Esquel (‘E’). Dark dots connected by lines in the figures in the left column represent the median of estimated locations for each twilight event. White dots show the median longitude and latitude of each non-breeding site. Orange polygons represent the error of each non-breeding site defined by the first and third quartiles of the median longitude and the latitude. When error is too small the polygons are not visible. Figures on the right column show changes in estimated longitude (top panels) and latitude (bottom panels). The black lines represent the median of the estimated location; dark shading represents the interquartile range and light gray shading the 95% credible interval. Red vertical lines represent equinoxes [35]. Base map and data from OpenStreetMap and OpenStreetMap Foundation.
(PDF)

S1 Table Number of light-level geolocators deployed and retrieved from *Elaenia albiceps* on Navarino Island and Esquel. We also indicate whether the geolocators were deployed as part of our study, a pilot study, or a long-term elaenia monitoring study.
(PDF)

S2 Table Individual migration schedule of *Elaenia albiceps* breeding in two sites within the South American temperate forests. Time is expressed in days. Data of Esquel breeding site obtained from Movebank Data Repository [28].
(PDF)
