# Supplementary material for: White-crested elaenias (Elaenia albiceps chilensis) breeding across Patagonia exhibit similar spatial and temporal movement patterns throughout the year
Source: PLoS One. 2024 Apr 18;19(4):e0299954. doi: 10.1371/journal.pone.0299954 (PMC11025734; doi:10.1371/journal.pone.0299954)
Supplement: S1 Fig — Dark dots connected by lines in the figures in the left column represent the median of estimated locations for each twilight event. White dots show the median longitude and latitude of each non-breeding site. Orange polygons represent the error of each non-breeding site defined by the first and third quartiles of the median longitude and the latitude. When error is too small the polygons are not visible. Figures on the right column show changes in estimated longitude (top panels) and latitude (bottom panels). The black lines represent the median of the estimated location; dark shading represents the interquartile range and light gray shading the 95% credible interval. Red vertical lines represent equinoxes [35]. Base map and data from OpenStreetMap and OpenStreetMap Foundation. (PDF) [file pone.0299954.s001.pdf]

H795 (N)	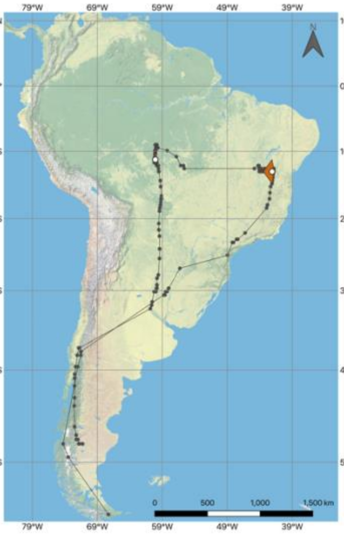	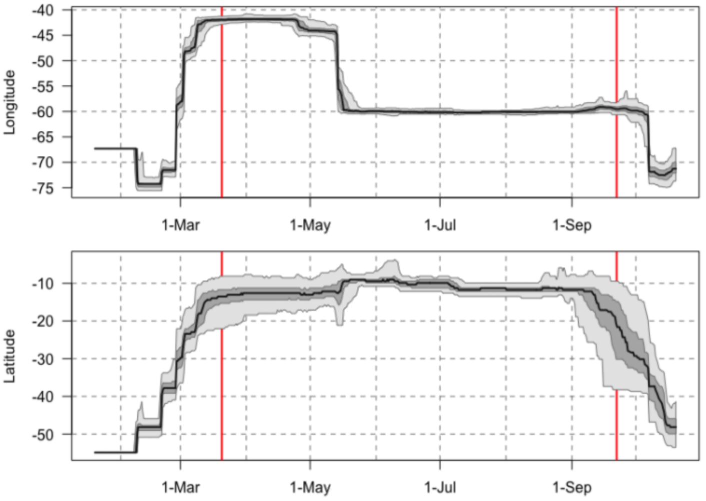
H796 (N)	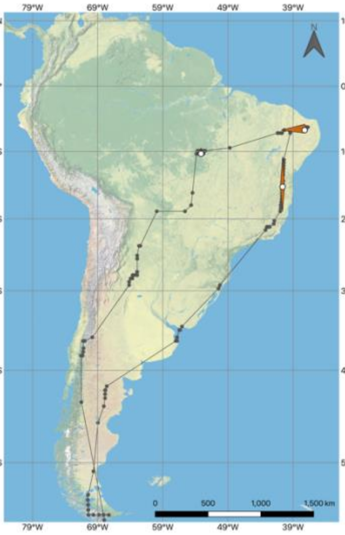	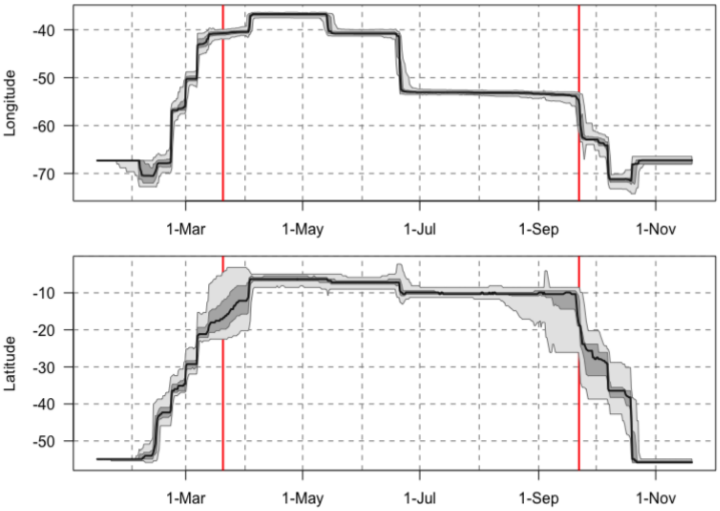
H820 (N)	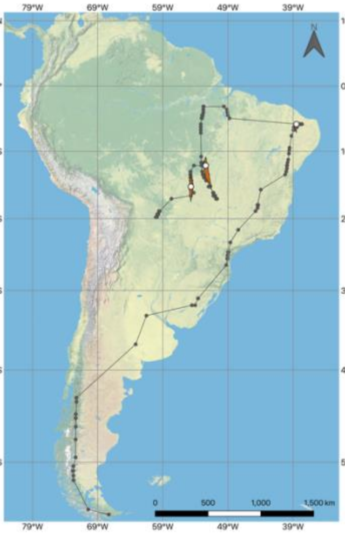	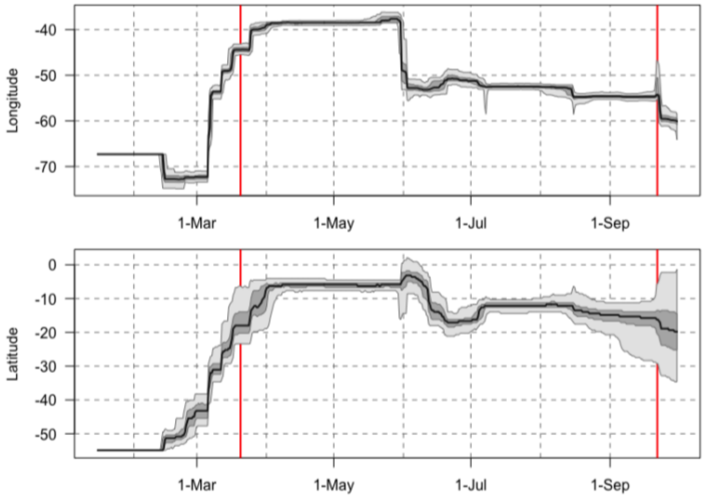

P438 (N)

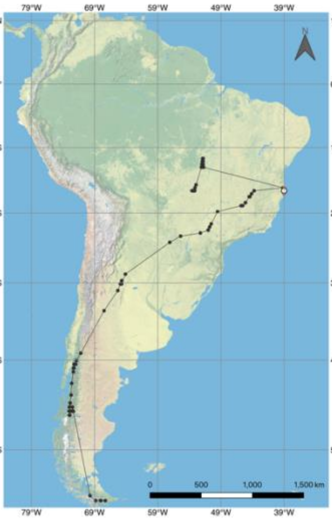

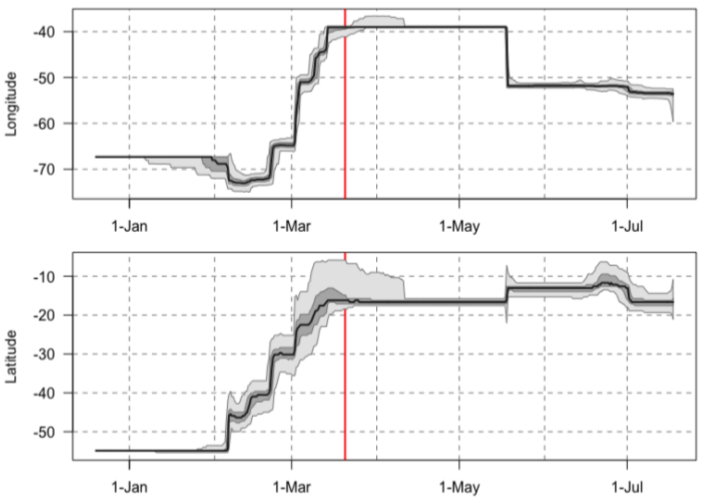

P453 (N)

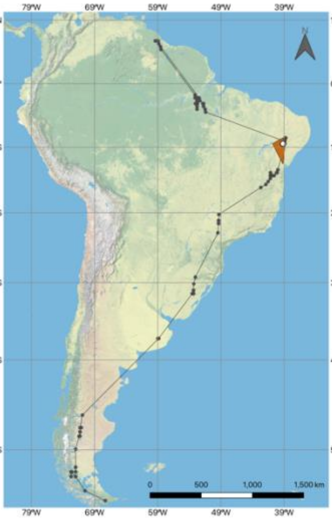

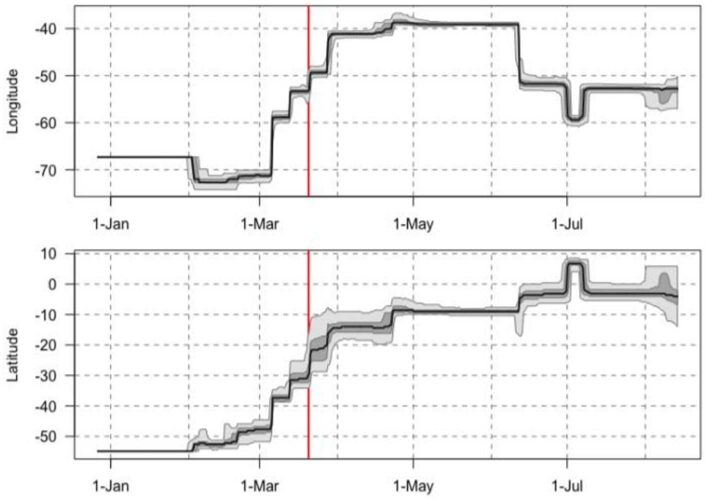

P461 (N)

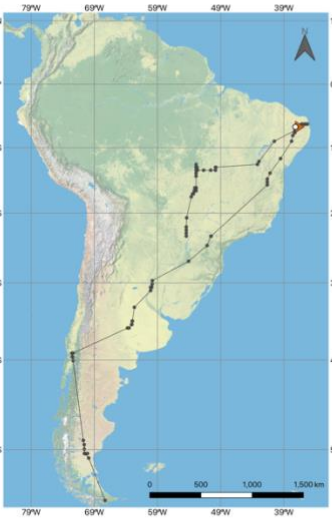

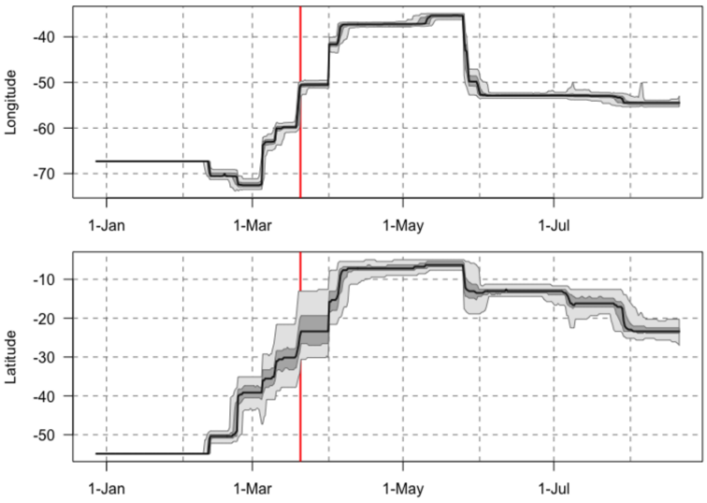

P472 (N)	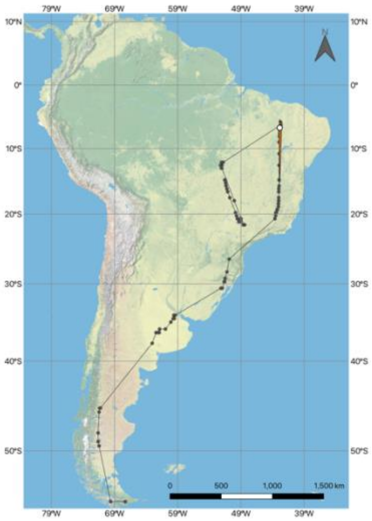	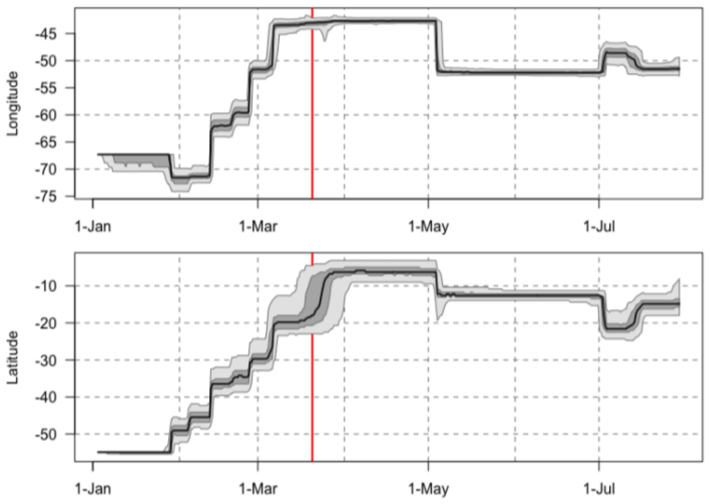
P481 (N)	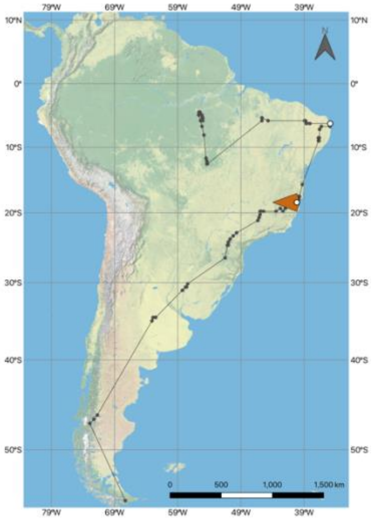	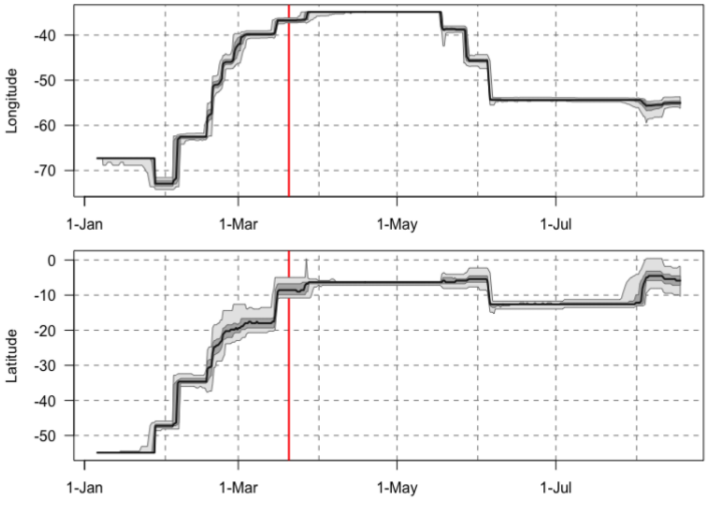
P488 (N)	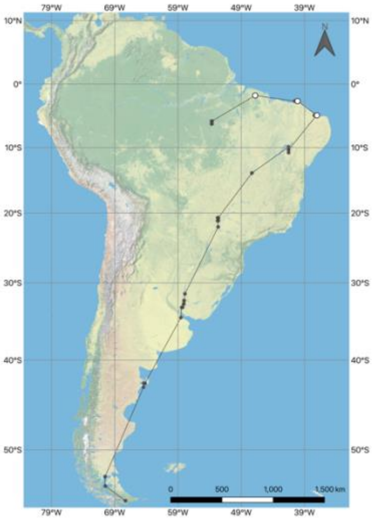	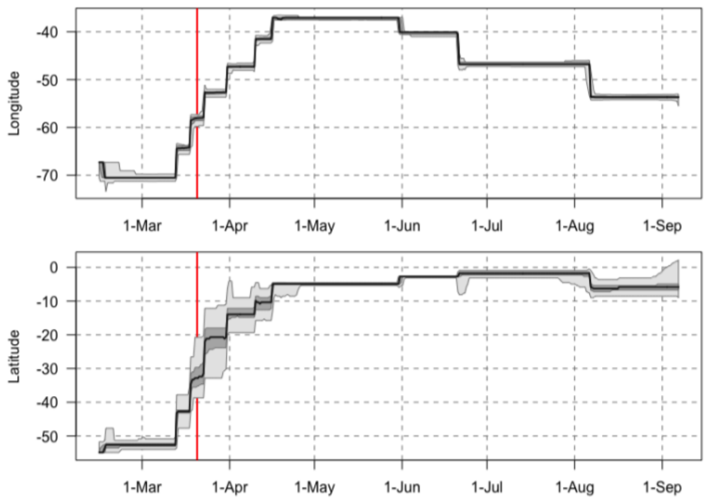

V859 (N)	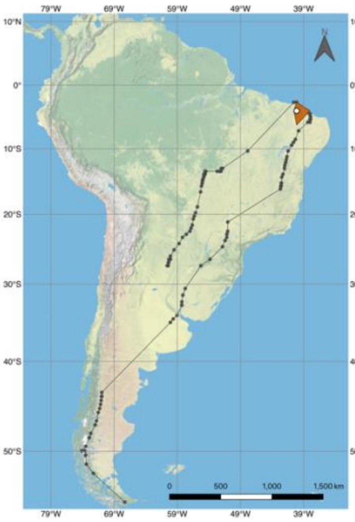	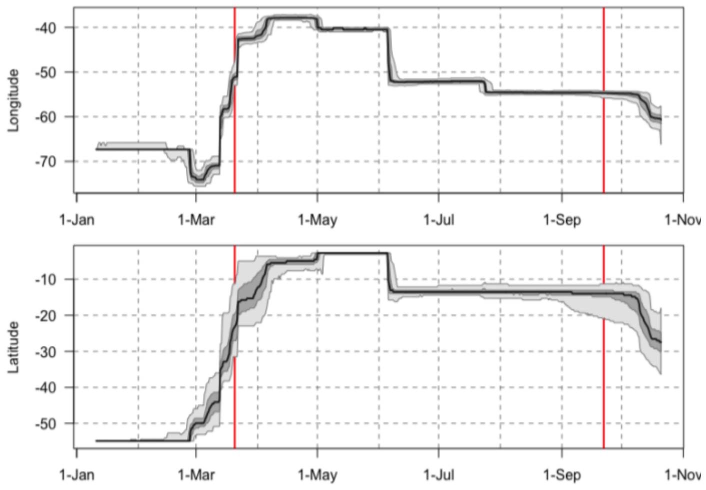
V860 (N)	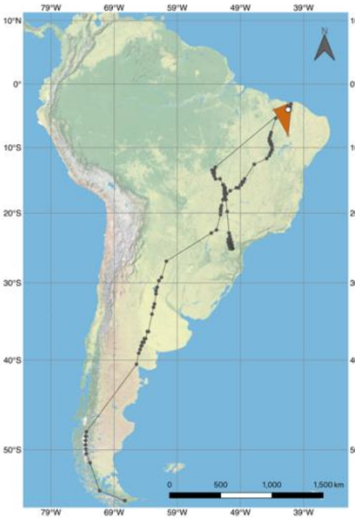	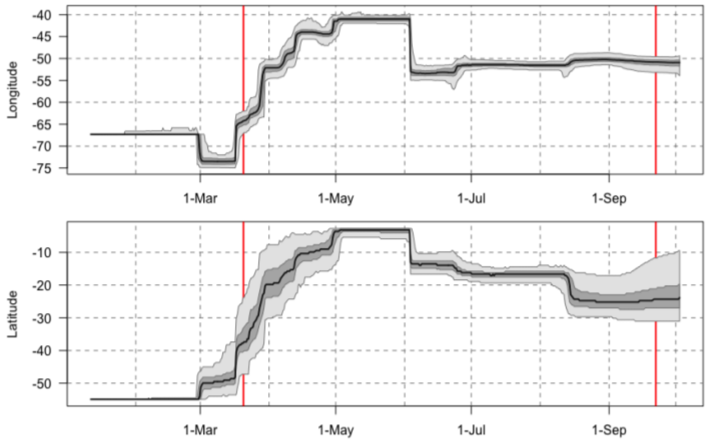
V873 (N)	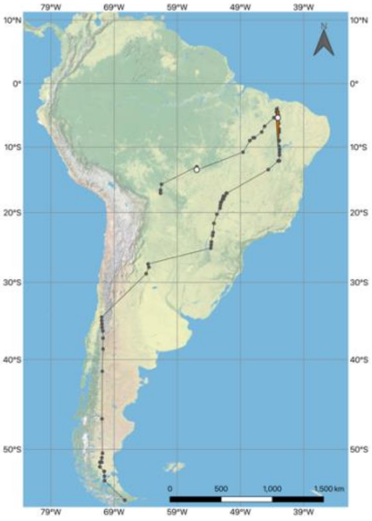	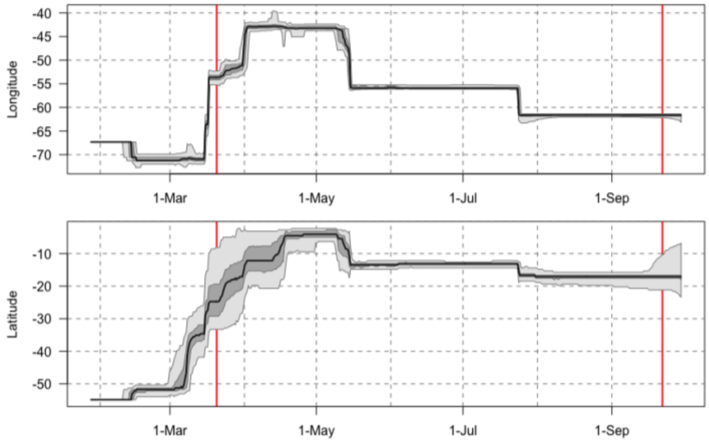

V876 (N)

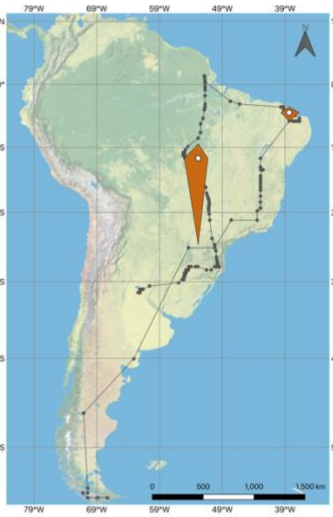

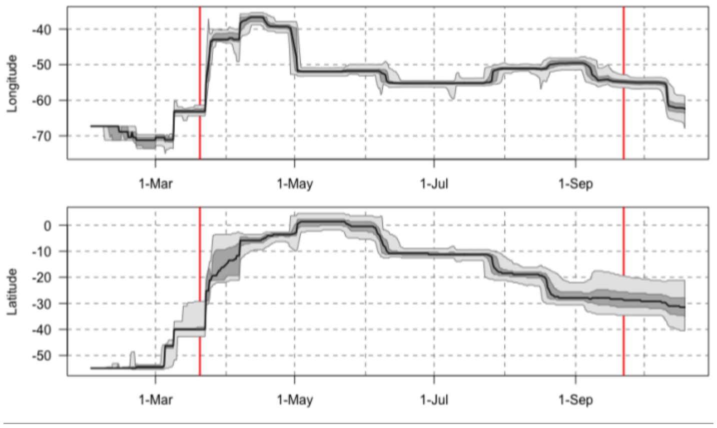

BC037 (N)

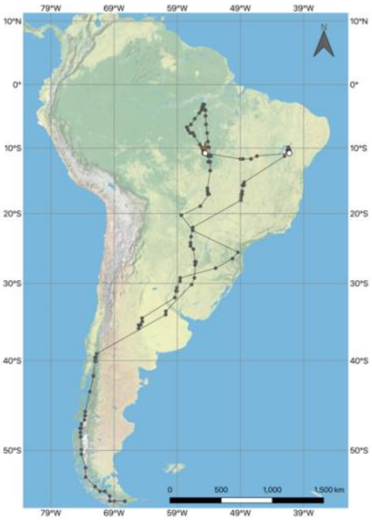

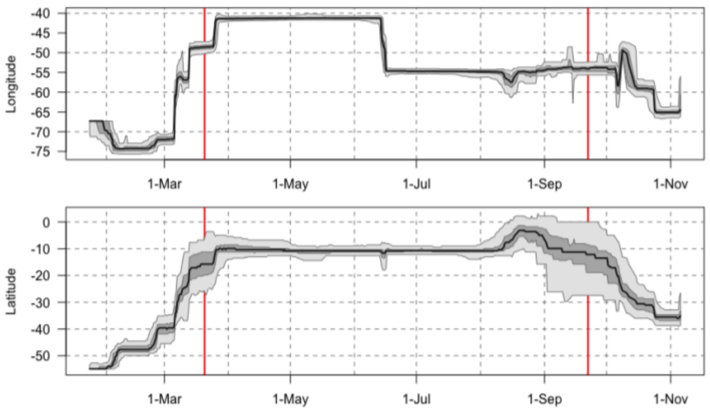

BJ354 (N)

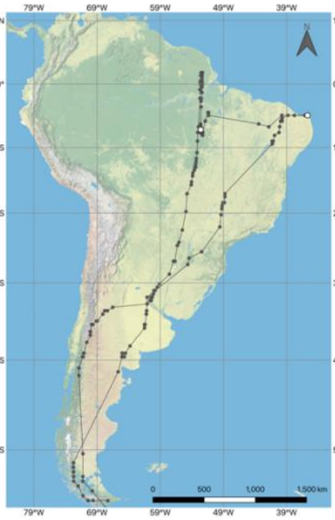

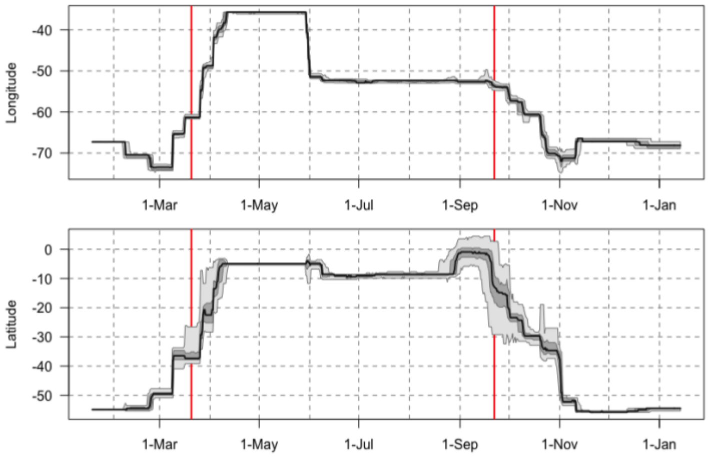

H750 (E)	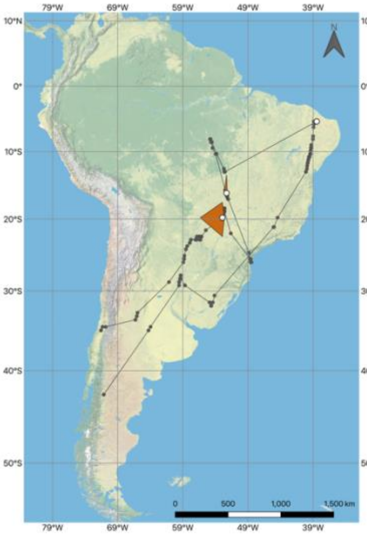	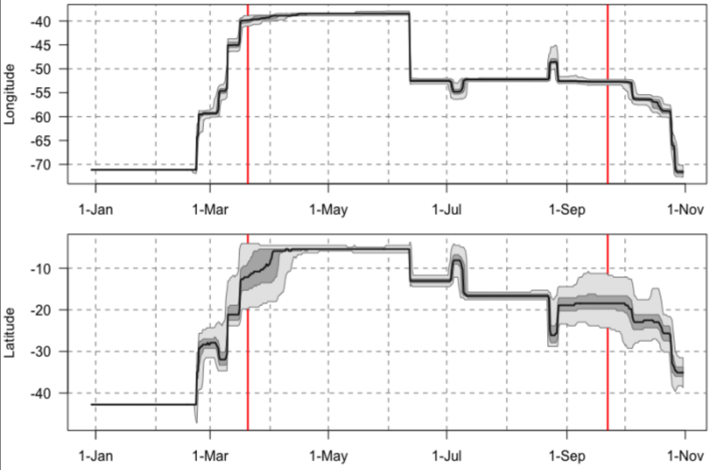
H753 (E)	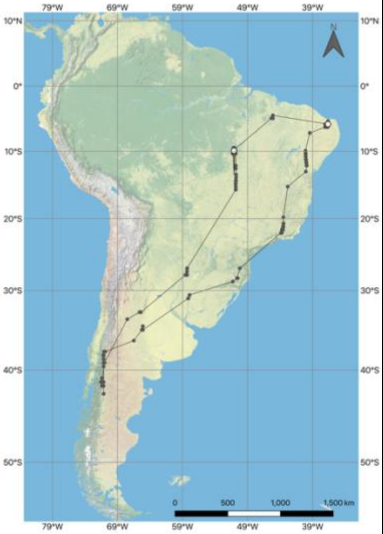	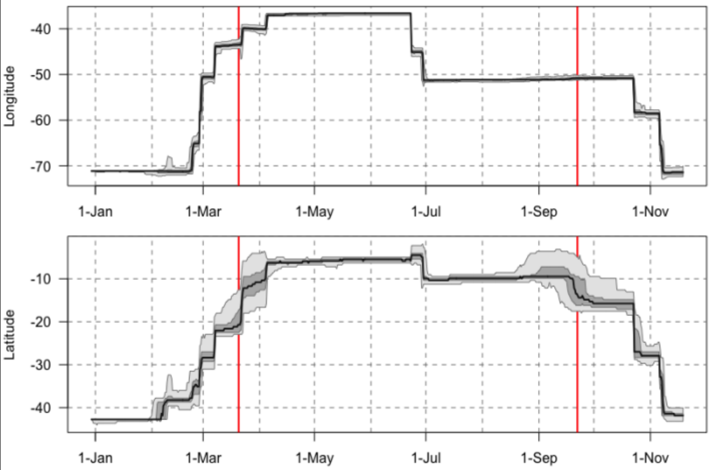
H755 (E)	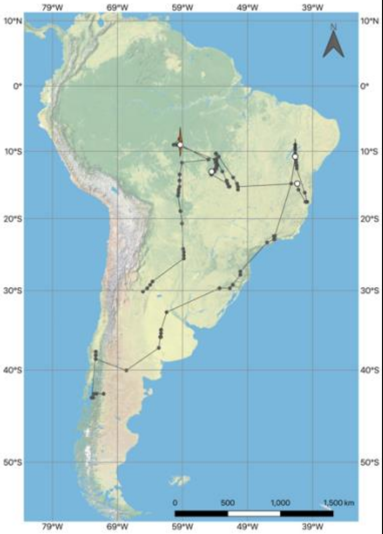	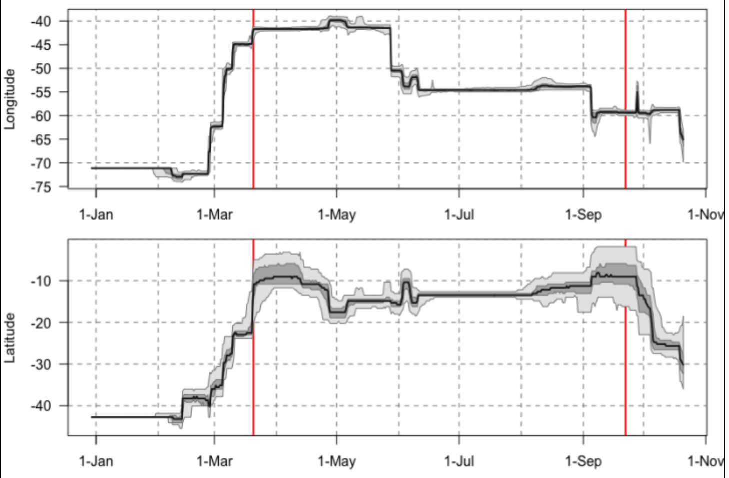

H757 (E)	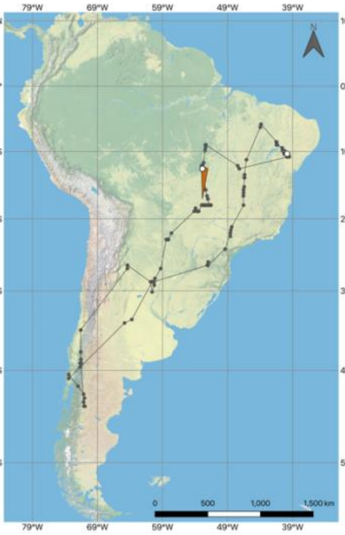	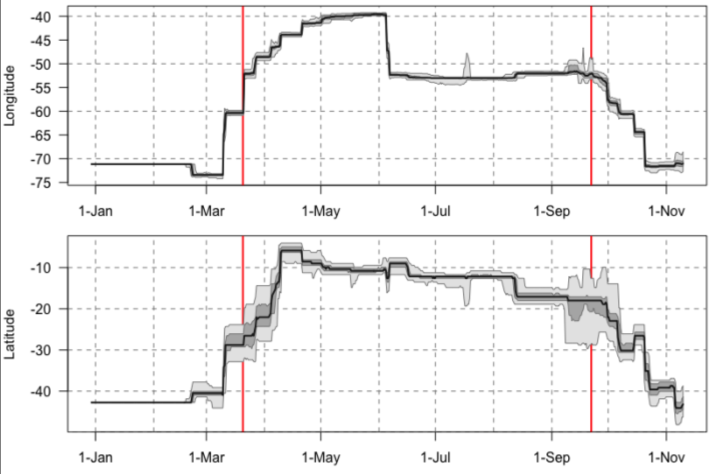
H760 (E)	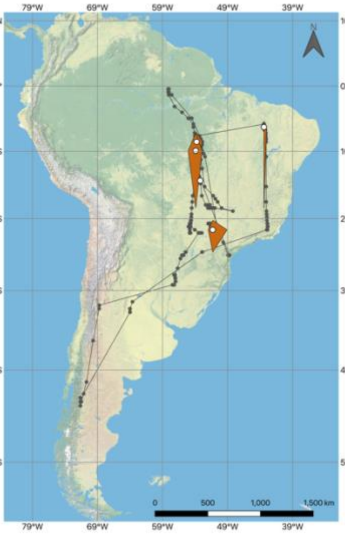	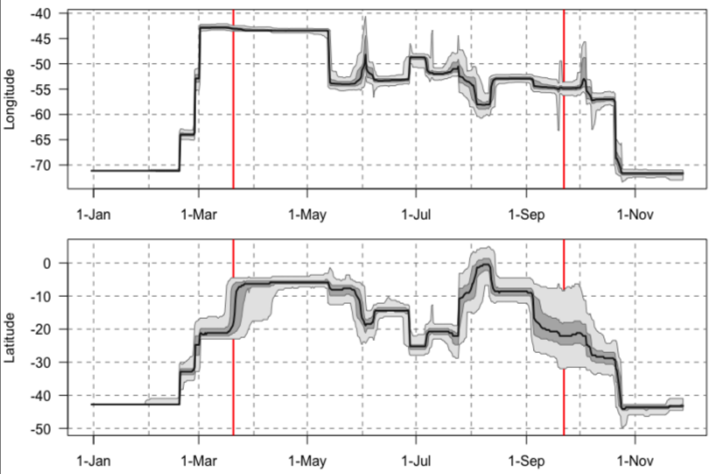
H762 (E)	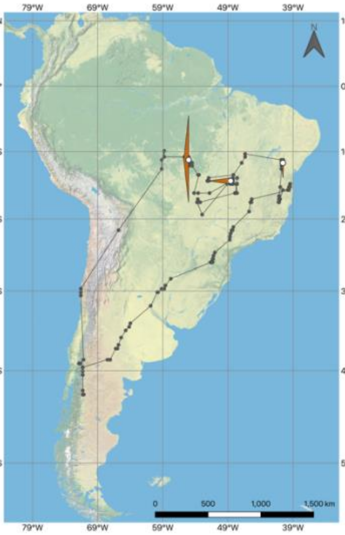	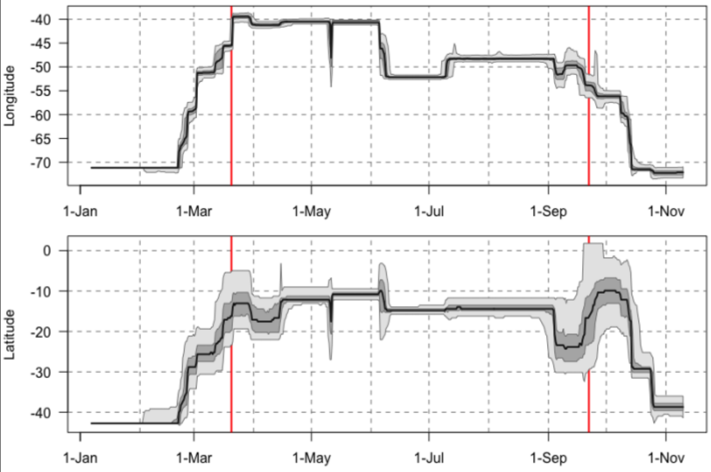

H764 (E)	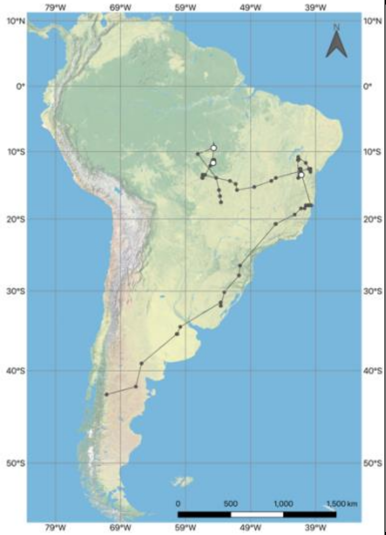	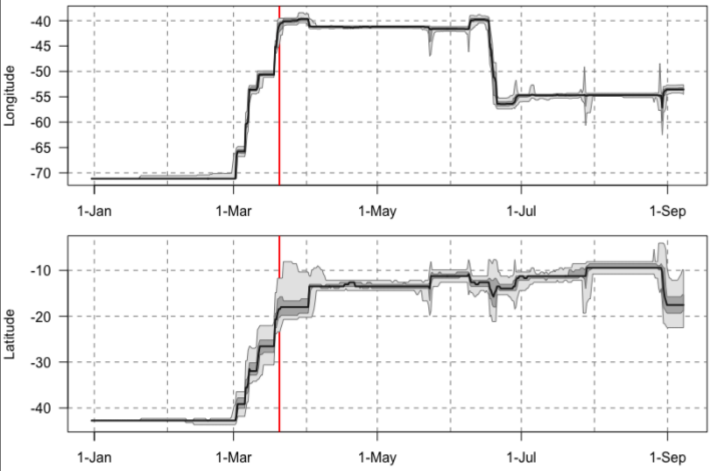
H769 (E)	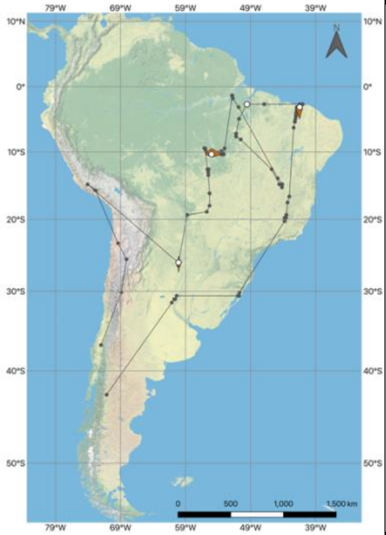	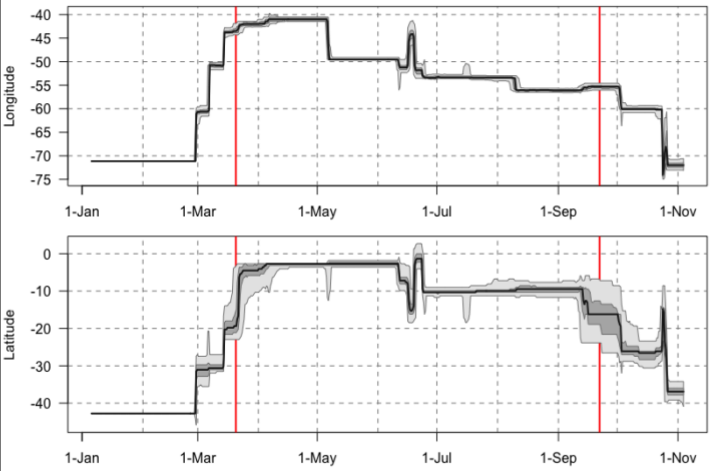
H773 (E)	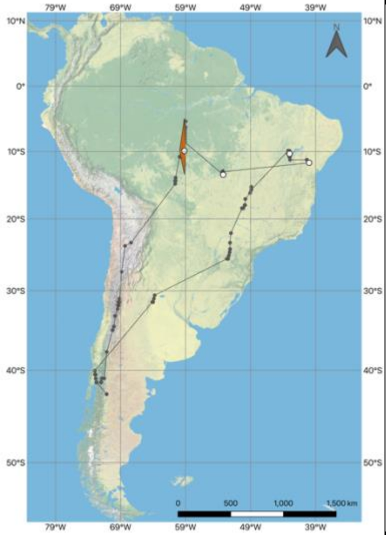	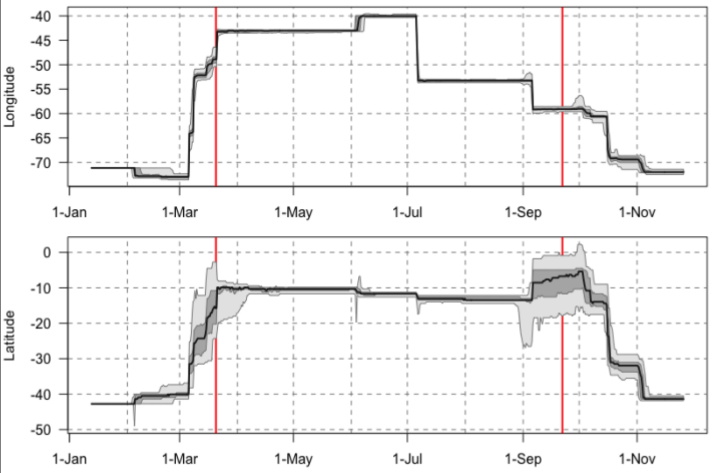

H775 (E)	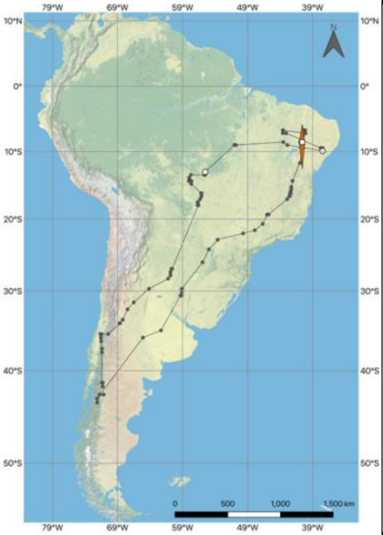	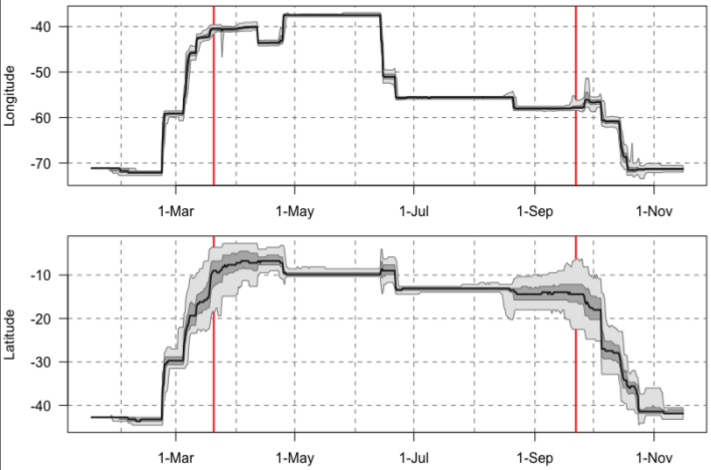
H777 (E)	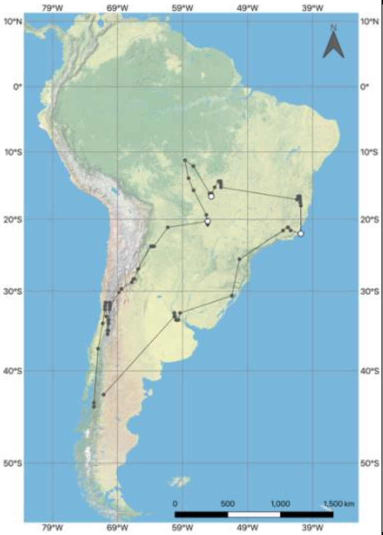	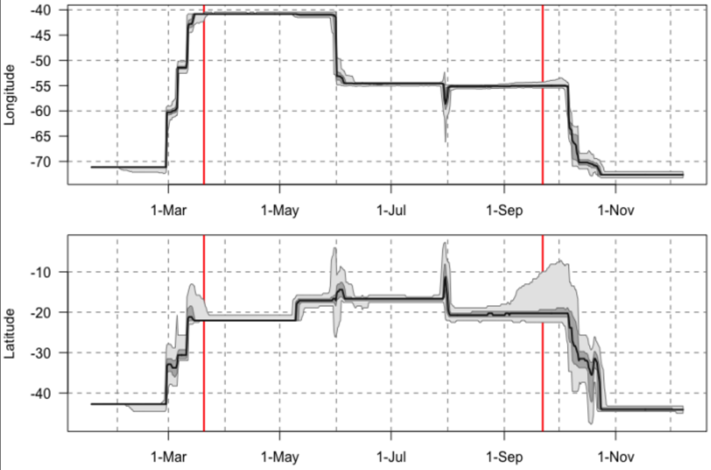
H780 (E)	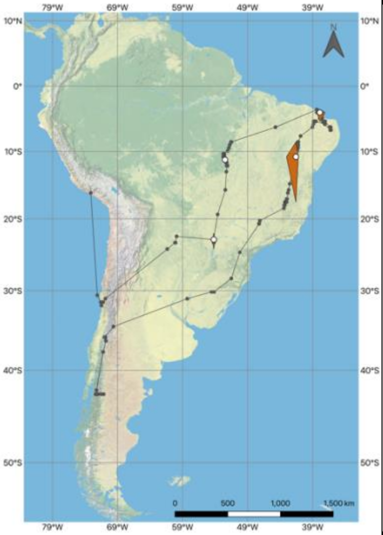	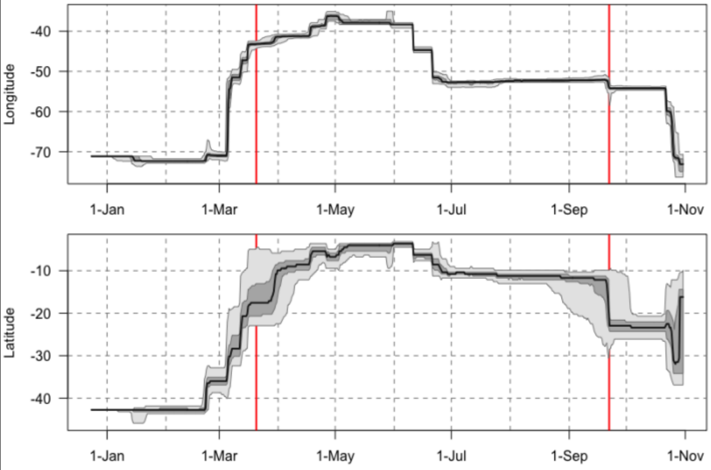

P859 (E)

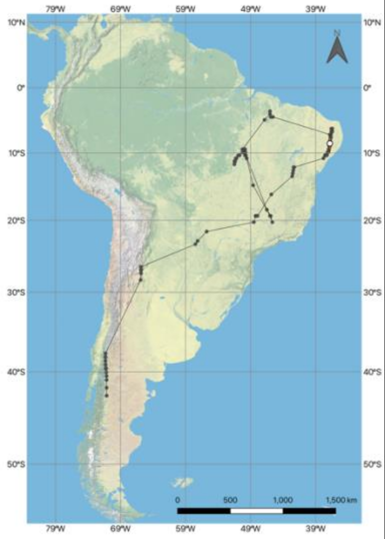

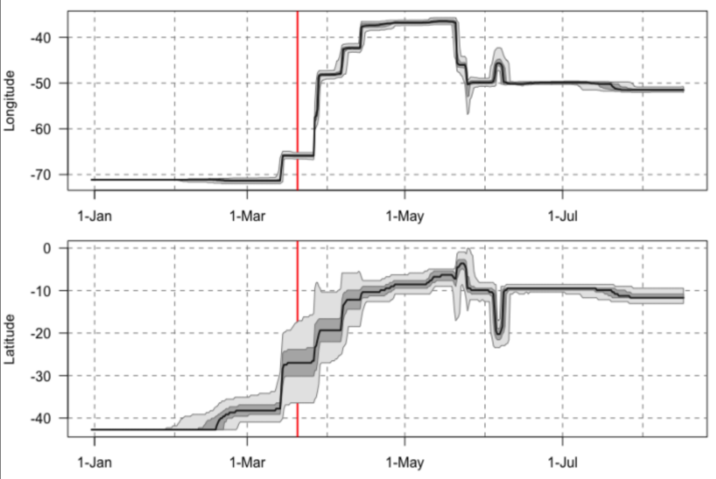

P868 (E)

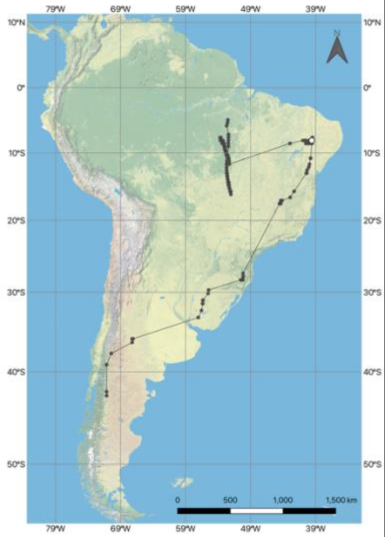

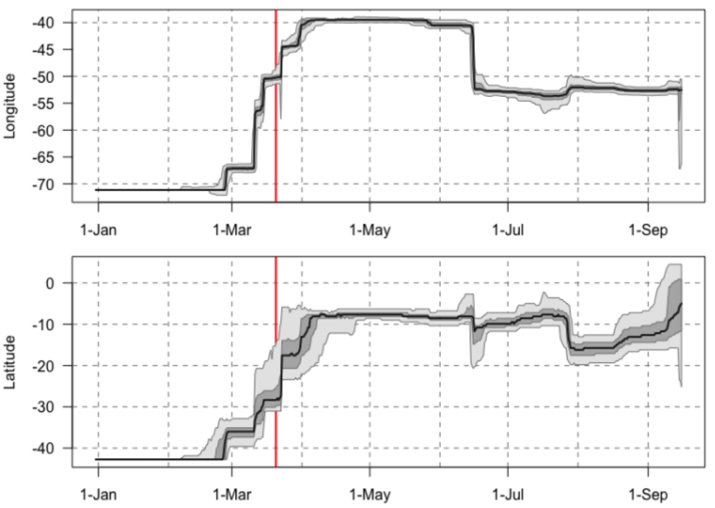

P872 (E)

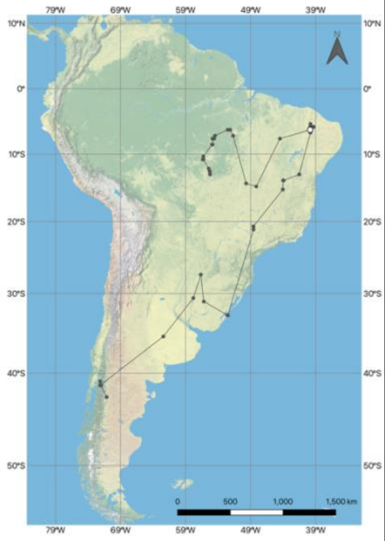

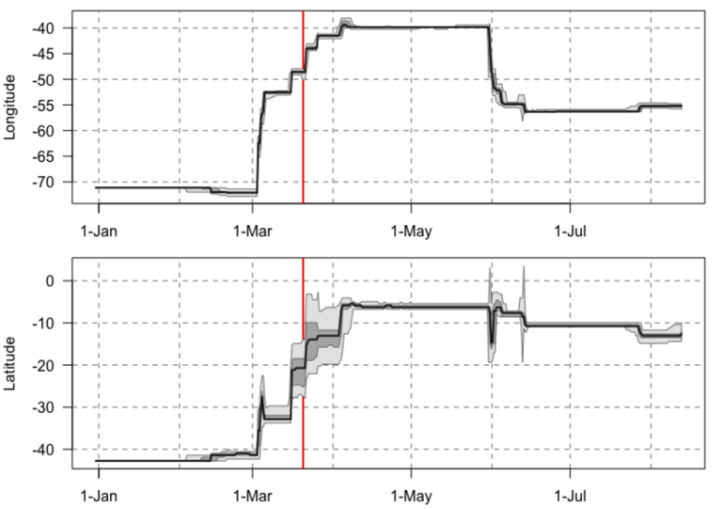

S1 Fig. Individual movement maps of elaenias breeding on Navarino Island (‘N’ in the ID) and Esquel (‘E’). Dark dots connected by lines in the figures in the left column represent the median of estimated locations for each twilight event. White dots show the median longitude and latitude of each non-breeding site. Orange polygons represent the error of each non-breeding site defined by the first and third quartiles of the median longitude and the latitude. When error is too small the polygons are not visible. Figures on the right column show changes in estimated longitude (top panels) and latitude (bottom panels). The black lines represent the median of the estimated location; dark shading represents the interquartile range and light gray shading the 95% credible interval. Red vertical lines represent equinoxes (Rakhimberdiev *et al.* 2017). Base map and data from OpenStreetMap and OpenStreetMap Foundation.
