# Supplementary material for: White-crested elaenias (Elaenia albiceps chilensis) breeding across Patagonia exhibit similar spatial and temporal movement patterns throughout the year
Source: PLoS One. 2024 Apr 18;19(4):e0299954. doi: 10.1371/journal.pone.0299954 (PMC11025734; doi:10.1371/journal.pone.0299954)
Supplement: S1 Table — We also indicate whether the geolocators were deployed as part of our study, a pilot study, or a long-term elaenia monitoring study. (PDF) [file pone.0299954.s002.pdf]

S1 Table. Number of light-level geolocators deployed and retrieved from *Elaenia albiceps* on Navarino Island and Esquel. We also indicate whether the geolocators were deployed as part of our study, a pilot study, or a long-term elaenia monitoring study.

Breeding site	Breeding season	Nos geolocators installed	Nos geolocator recovered	Recovery rate	Study
Navarino	2013/2014	39	3	7.7%	Pilot study
	2014/2015	57	6	10.5%	Our study
	2015/2016	60	6	10.0%	Our Study
	2016/2017	33	3[1]	9.1%	Long-term study
	2017/2018	12	4[2]	33.3%	Long-term study
Esquel	2013/2014	35	12	34.3%	Bravo et al. 2017
	2014/2015	10	3	30.0%	Bravo et al. 2017

[1] Only one of these geolocators where included in this study: BC037
[2] Only one of these geolocators where included in this study: BJ354. This device recorded light data for 2 years. However, to maintain consistency among all samples, we analyzed the data from only the first annual cycle.

Bravo, S.P., Cueto, V.R. & Gorosito, C.A. 2017. Migratory timing, rate, routes and wintering areas of White-crested Elaenia (Elaenia albiceps chilensis), a key seed disperser for Patagonian forest regeneration. *PLoS One* 12: e0170188.
