# Supplementary material for: White-crested elaenias (Elaenia albiceps chilensis) breeding across Patagonia exhibit similar spatial and temporal movement patterns throughout the year
Source: PLoS One. 2024 Apr 18;19(4):e0299954. doi: 10.1371/journal.pone.0299954 (PMC11025734; doi:10.1371/journal.pone.0299954)
Supplement: S2 Table — Time is expressed in days. Data of Esquel breeding site obtained from Movebank Data Repository [28]. (PDF) [file pone.0299954.s003.pdf]

S2 Table. Individual migration schedule of *Elaenia albiceps* breeding in two sites within the South American temperate forests. Time is expressed in days. Data of Esquel breeding site obtained from Movebank Data Repository (Cueto & Bravo 2017)

			**Fall migration**		**Non-breeding season**								**Spring migration**	
**Breeding site**	**ID**	**Year**	**Departure date**	**Arrival date**	**No. non-breeding sites**[1]	**Arrival at 1st region**[2]	**Departure from 1st region**	**Days at 1st region**	**Arrival at 2nd region**	**Departure from 2nd region**	**Days at 2nd region**	**Overall non-breeding season duration**	**Departure date**	**Arrival date**
Navarino	H795	2014	8-Feb	8-Mar	2	8-Mar	13-May	66	16-May	6-Oct	143	212	6-Oct	ND[3]
Navarino	H796	2014	21-Feb	11-Mar	3	11-Mar	20-Jun	101	21-Jun	21-Sep	92	194	21-Sep	19-Oct
Navarino	H820	2014	12-Feb	25-Mar	3	25-Mar	29-May	65	1-Jun	21-Sep	112	180	21-Sep	ND
Navarino	P438	2015	5-Feb	14-Mar	ND	14-Mar	18-May	65	ND	ND	ND	ND	ND	ND
Navarino	P543	2015	5-Mar	28-Mar	ND	28-Mar	11-Jun	75	ND	ND	ND	ND	ND	ND
Navarino	P461	2015	11-Feb	5-Apr	ND	5-Apr	25-May	50	ND	ND	ND	ND	ND	ND
Navarino	P472	2015	28-Jan	6-Mar	ND	6-Mar	3-May	58	ND	ND	ND	ND	ND	ND
Navarino	P481	2015	27-Jan	22-Feb	ND	22-Feb[4]	18-May	85	ND	ND	ND	ND	ND	ND
Navarino	P488	2015	12-Mar	16-Apr	ND	16-Apr	20-Jun	65	ND	ND	ND	ND	ND	ND
Navarino	V859	2016	12-Mar	21-Mar	ND	21-Mar	5-Jun	76	ND	ND	ND	ND	ND	ND
Navarino	V860	2016	16-Mar	12-Apr	ND	12-Apr	3-Jun	ND	ND	ND	ND	ND	ND	ND
Navarino	V873	2016	15-Mar	1-Apr	ND	1-Apr	14-May	43	14-May	ND	ND	ND	ND	ND
Navarino	V876	2016	17-Feb	6-Apr	2	6-Apr	30-Apr	24	1-May	10-Oct	162	187	10-Oct	ND
Navarino	BC037	2017	5-Mar	25-Mar	2	25-Mar	13-Jun	80	15-Jun	5-Oct	112	194	5-Oct	ND
Navarino	BJ354	2018	8-Mar	4-Apr	2	4-Apr	29-May	55	31-May	20-Oct	142	199	20-Oct	10-Nov
**Average**			**22-Feb**	**24-Mar**	**2**	**24-Mar**	**27-May**	**65**	**28-May**	**4-Oct**	**127**	**194**	**4-Oct**	**30-Oct**
Esquel	H750	2014	21-Feb	16-Mar	3	16-Mar	11-Jun	87	11-Jun	24-Oct	135	222	24-Oct	ND
Esquel	H753	2014	22-Feb	4-Apr	2	4-Apr	22-Jun	79	29-Jun	22-Oct	115	201	22-Oct	6-Nov
Esquel	H755	2014	12-Feb	20-Mar	4	20-Mar	27-May	68	10-Jun	27-Sep	109	191	27-Sep	ND
Esquel	H757	2014	21-Feb	1-May	2	1-May	4-Jun	34	6-Jun	29-Sep	115	151	29-Sep	22-Oct
Esquel	H760	2014	18-Feb	1-Mar	5	1-Mar	13-May	73	13-May	3-Oct	143	216	3-Oct	23-Oct
Esquel	H762	2014	20-Feb	21-Mar	3	21-Mar	5-Jun	76	5-Jun	8-Oct	125	201	8-Oct	ND
Esquel	H764	2014	2-Mar	2-Apr	3	2-Apr	23-May	51	20-Jun	29-Aug	70	149	29-Aug	ND
Esquel	H769	2014	27-Feb	14-Mar	4	14-Mar	11-Jun	89	23-Jun	23-Oct	122	223	23-Oct	ND
Esquel	H773	2014	5-Mar	21-Mar	4	21-Mar	6-Jul	107	6-Jul	15-Oct	101	208	15-Oct	17-Oct
Esquel	H775	2014	21-Feb	11-Mar	3	11-Mar	13-Jun	94	21-Jun	20-Aug	60	162	20-Aug	23-Oct
Esquel	H777	2014	27-Feb	14-Mar	3	14-Mar	31-May	78	1-Jun	5-Oct	126	205	5-Oct	11-Oct
Esquel	H780	2014	21-Feb	16-Mar	4	16-Mar	10-Jun	86	21-Jun	21-Oct	122	219	21-Oct	ND
Esquel	P859	2015	14-Mar	14-Apr	ND	14-Apr	20-May	36	ND	ND	ND	ND	ND	ND
Esquel	P868	2015	25-Feb	31-Mar	ND	31-Mar	15-Jun	76	ND	ND	ND	ND	ND	ND
Esquel	P872	2015	12-Feb	6-Apr	ND	6-Apr	31-May	55	ND	ND	ND	ND	ND	ND
**Average**			**24-Feb**	**25-Mar**	**3**	**25-Mar**	**6-Jun**	**73**	**14-Jun**	**2-Oct**	**110**	**194**	**2-Oct**	**22-Oct**

[1] "Non-breeding site" refers to a discrete site-specific non-breeding location that lasted at least 20 days. This is different from "non-breeding region", which we use throughout the manuscript to refer to the two large regions used sequentially by all elaenias in this study. If a geolocator stopped recording data before the initiation of spring migration, we did not report the number of non-breeding sites used by that individual.

[2] For schedule estimation purposes, if an individual used more than one non-breeding site within a non-breeding region, we grouped these sites to estimate arrival and departure date, as well as length of stay.

[3] ND = No data. It could not be estimated because the geolocator ran out of battery.

[4] This individual's first sedentary period (where it stayed 20 days) was located in central-eastern Argentina, in the Humid Pampas ecoregion. This area is along the fall migration route for many other elaenias breeding in either Esquel or Navarino. In addition, it is located over 3,000 km south of the average first non-breeding region of the other 29 individuals. After stopping at this site, the next long sedentary period was in the Bahia Coastal Forests in Brazil, where the bird spent 20 days, and which is within the first non-breeding region occupied by the rest of the tracked birds. We therefore treated the sedentary period in Argentina as a fall migration stopover and considered the second sedentary period in Brazil as the first non-breeding site for this individual.
